# Roll-To-Roll Coated Metal–Organic Framework (MOF)-Fabric-Based Filters for Particulate Matter Filtration and Chemical Warfare Agent Degradation

**DOI:** 10.1021/acsami.6c03444

**Published:** 2026-05-27

**Authors:** Ankit Dhakal, Lauren Hernon, Sangeun Jung, Emily Beyer, Sean Robinson, Luke Huelsenbeck, Prince Verma, Bala Mulloth, Gregory W. Peterson, Gaurav Giri

**Affiliations:** † Department of Chemical Engineering, 2358 University of Virginia, Charlottesville, Virginia 22904, United States; ‡ Frank Batten School of Leadership and Public Policy, 2358 University of Virginia, Charlottesville, Virginia 22904, United States; § U.S. Army Combat Capabilities Development Command Chemical Biological Center, Aberdeen Proving Ground, Aberdeen, Maryland 21010, United States

**Keywords:** metal−organic framework, UiO-66, filtration, chemical warfare agent degradation

## Abstract

Metal–organic frameworks (MOFs) have been widely explored as adsorbents and filtration materials for gases, small molecules, and biomolecules. More recently, integrating MOFs into textiles has enabled the development of MOF-fabrics for particulate matter (PM) filtration and chemical warfare agent (CWA) degradation. However, the scalability of creating MOF-fabrics for large-area, high-throughput production is a significant issue preventing the widespread adoption of MOF-fabrics for practical implementation. Here, we utilize a novel aqueous method to rapidly synthesize zirconium-MOFs UiO-66 and UiO-66-NH_2_ on the surfaces of polyester and cotton fabrics, using multiple cycles of a sequential dip-coating (SQD) technique combined with a roll-to-roll-compatible process. UiO-66-polyester MOF-fabric masks demonstrated enhanced PM filtration and met ASTM F3502 Level 1 performance criteria, achieving filtration efficiencies above 20% with a pressure drop below 15 mm H_2_O for 0.3 μm particles. The best-performing MOF-fabric mask reached a filtration efficiency of 39.7%. The UiO-66-polyester masks also exhibited low cytotoxicity and maintained performance even after 25 washes, supporting their use as reusable facial coverings. In parallel, UiO-66-NH_2_-fabric demonstrated enhanced CWA degradation performance, with UiO-66-NH_2_-cotton degrading 82% and 89% of the nerve agents GD and VX, respectively, within 24 h. Overall, this work establishes a scalable pathway for the synthesis of high-performance MOF-fabrics while enabling broader application of these materials in practical protective technologies.

## Graphical abstract

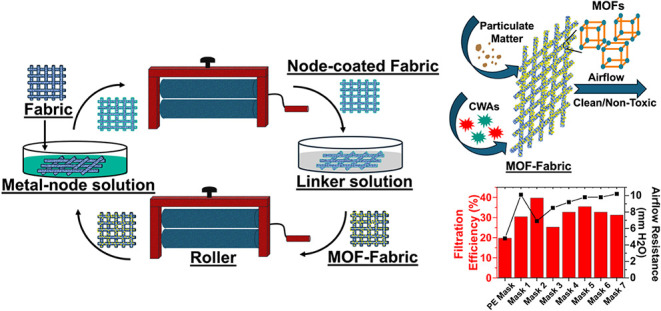

## Introduction

Metal–organic frameworks (MOFs) are highly porous materials composed of metal ions or clusters coordinated with organic linkers.^1,2^ Their exceptionally high surface areas, tunable pore sizes, and versatile chemical functionalization make them attractive for separation and filtration technologies. However, the intrinsic brittleness of crystalline MOFs presents a key barrier to translating their properties into practical, real-world applications.^3–6^ Integrating MOFs with flexible substrates such as filters and fabrics offers a practical route to overcome this limitation, yielding composites that combine the porosity and functionality of the MOF with the durability and processability of the substrate.^7–9^ In one such example, the integration of MOFs into textile fabrics has enabled their use in particulate matter (PM) filtration, where the MOF surface charge facilitates particle rejection.^10,11^ In addition, MOF-fabrics have been developed as smart textiles with sensing or chemical warfare agent (CWA) detoxification functions, positioning them for multifunctional applications.^8,12,13^

To evaluate the role of MOFs in PM filtration, it is important to consider the underlying particle-capture mechanisms in fibrous media, where multiple filtration mechanisms operate simultaneously. Larger particles (>1 μm) are blocked via inertial impaction, as particle momentum prevents them from following the deflected airstream around a fiber, causing direct collision with the fiber surface.^14^ On the other hand, the motion of ultrafine particles (<0.1 μm) is dominated by Brownian diffusion, where random motion of the particles causes them to deviate from streamlines and contact the fiber surface.^15^ Since the diffusion coefficient scales inversely with particle diameter, smaller particles diffuse more rapidly and are therefore blocked more efficiently. Particle interception is least efficient for intermediate particle sizes around 0.3 μm, where particle inertia is insufficient to cause fiber impaction and particle size is too large for Brownian motion to produce significant deviation from streamlines.^15^ This size range is defined as the most penetrating particle size (MPPS) and serves as a benchmark in PM filtration design.^14–16^

Incorporating MOFs onto the fabric surface introduces additional surface-mediated interactions that improve blockage of MPPS (PM_0.3_) particles (Figure 1a). The open metal sites and functional groups within MOFs enable electrostatic interaction with charged particles.^17,18^ The MOF coating on the fiber surface also increases surface roughness and available surface area, which enhances interception and adsorption.^19,20^ Several studies have demonstrated the varied potential of MOF-fabrics in the laboratory. Yoo et al. demonstrated that cotton fabrics coated with four different Zr-based MOFs achieved up to 73% filtration efficiency for PM_2.5_.^21^ Similarly, Zhang et al. also tested the PM filtration efficiency of four MOFs (ZIF-8, UiO-66-NH_2_, MOF-199, and Mg-MOF-74). These MOFs were dispersed into fibers to study their interactions with pollutants, and the study showed that the ZIF-8/polyacrylonitrile (PAN) MOF filter achieved up to 88% filtration efficiency for PM_2.5_.^17^ These results collectively highlight that integrating MOFs into fibrous substrates can significantly enhance their PM filtration efficiency.

**Figure 1:**
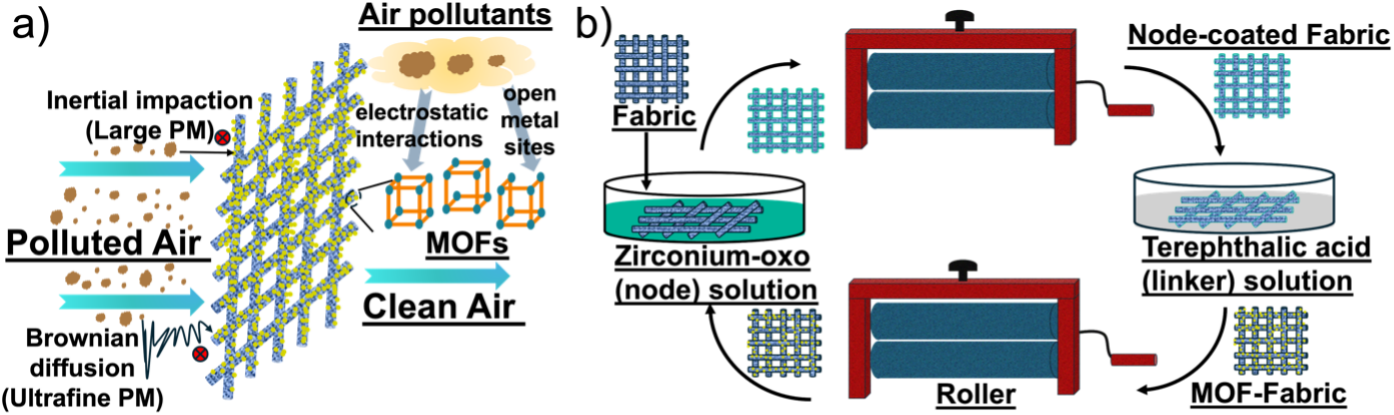
a) Schematic illustration of PM filtration by MOF-fabrics. PM-laden air (left) passes through the MOF-coated textile, where large particles are blocked via inertial impaction, and ultrafine particles, that move via Brownian diffusion, are stopped. The cuboid structures represent MOFs that help intercept MPPS (PM ∼ 0.3 μm) via electrostatic interactions and physical adsorption. b) Flow diagram of the SQD process showing one complete cycle. In each cycle, the fabric is first immersed in the zirconium-oxo (Zr-oxo) node solution, then passed through the rollers to remove excess liquid. It is then dipped into the organic linker solution, followed by another rolling step to remove excess solution. One complete sequence constitutes an SQD cycle. The MOF layer (yellow dots) builds up progressively with each cycle; a total of 8 SQD cycles were performed.

However, to effectively evaluate and compare the filtration performance of different filters or fabrics, a standardized testing framework is necessary to assess filtration under realistic conditions. In this regard, following the COVID-19 pandemic, the American Society for Testing and Materials (ASTM) International introduced the F3502 barrier face-covering specification to establish benchmarks for nonmedical masks, with performance defined by both filtration efficiency and breathability. The ASTM F3502 defines two performance levels (Level 1 and Level 2), with Level 2 corresponding to higher filtration efficiency and lower airflow resistance, as summarized in Table S1.^22^ Because ASTM F3502 considers both capture efficiency and breathability, it has become a widely accepted reference for assessing the practical performance of filtration materials. Nevertheless, despite the relevance of standardized testing protocols, relatively few academic studies, particularly those involving MOF-fabrics, have reported results based on standardized testing methods, with most prior research employing nonstandardized testing methodologies that limit direct comparison.^23–25^

In addition to PM filtration, incorporating MOFs into fabrics has been shown to improve the degradation of CWAs.^13,26^ Balasubramanian et al. incorporated Zr-MOFs (UiO-66 and UiO-66-NH_2_) into cotton fabrics and demonstrated enhanced detoxification performance against the CWA simulant, methyl-paraoxon (DMNP), with maximum conversions of 88.9% and 90.68% for UiO-66 and UiO-66-NH_2_ MOF-fabrics, respectively.^27^ However, most existing syntheses rely on toxic solvents such as dimethylformamide (DMF) and require long reaction times, limiting their translation to industrial-scale production.^28^ Despite the advantages of MOF-textiles, large-scale synthesis remains challenging due to cost, scalability, and environmental sustainability concerns.

Recently, advancements in rapid, aqueous MOF synthesis have enabled the high-throughput, eco-friendly production of MOF-coated fabrics.^28,29^ Compared to conventional approaches that rely on DMF, water-based synthesis has a lower environmental footprint and is better suited to large-scale production. Huelsenbeck et al. demonstrated the rapid fabrication of several prototypical MOF-coated fabrics, including UiO-66, UiO-66-NH_2_, ZIF-L, and HKUST-1, using a sequential dip-coating (SQD) process.^29^ The SQD process involves repeatedly immersing textiles in the metal node and the organic linker solutions, respectively, allowing diffusion of the precursors into and onto the textile fibers (Figure 1b). Since the reaction between the node and the linker occurs only at the fiber surface, MOFs are grown as firmly attached coatings.^8,30^ These MOF-fabrics, particularly UiO-66-NH_2_ on cotton, achieved high performance for PM filtration, with filtration efficiencies of ∼80% for PM_1–4_.^29^ Although this study established a proof-of-concept for SQD-derived MOF-fabrics on an inch^2^ scale, the extension of this approach to large-area textiles, practical mask fabrication, and advanced functions such as CWA degradation has not yet been explored.

In this work, we report the fabrication of large-area (9″ × 6″) MOF-textiles composed of UiO-66 on polyester using the SQD method. Polyester fabric was selected for its wide availability, durability, and favorable breathability, all of which are essential for practical face coverings. In addition, polyester can retain more static charge compared to other natural fibers, which is crucial for enhanced PM filtration.^31^ UiO-66, a zirconium (Zr)-based MOF, made of Zr-oxo clusters as nodes and terephthalic acid as the linker, is known for its stability in aqueous environments.^32^ In addition, UiO-66 is less toxic compared to other MOFs, which is crucial if the MOF-fabric is to be used as a reusable facial covering.^33,34^ We demonstrate that UiO-66-polyester composites can be processed into full-sized masks that meet the ASTM F3502 Level 1 standards, achieving filtration efficiencies of up to ∼39% for 0.3 μm particles while maintaining breathability, as measured by a pressure drop across the fabric of 6.9 mm H_2_O at an airflow rate of 85 ± 4 L/min. Additionally, the UiO-66-polyester masks maintain their performance even after multiple washing-drying cycles (up to 25 times), demonstrating their reusability. Beyond filtration, we extended the SQD approach to synthesize UiO-66-NH_2_ on the surface of polyester and cotton. We then studied the performance of UiO-66-NH_2_-fabrics in the catalytic degradation of CWAs such as GD (3,3-dimethyl-2-butyl methylphosphonofluoridate, or Soman), HD (2,2′-dichloroethyl sulfide, or mustard), and VX (*O*-ethyl *S*-2-(diisopropylamino)­ethyl methyl phosphonothioate). We demonstrate the successful formation of UiO-66-NH_2_ on the surfaces of polyester and cotton and show that the MOF-fabric exhibits enhanced degradation of GD and VX. Within 24 h, 82% and 89% of GD and VX are decomposed by the UiO-66-NH_2_-cotton fabric. Altogether, this work establishes a scalable SQD process for producing MOF-textiles, demonstrating their applicability for PM filtration and CWA detoxification, and positioning them as a practical platform for next-generation protective fabrics.

## Materials

Zirconyl chloride octahydrate (ZrOCl_2_·8H_2_O), glacial acetic acid, terephthalic acid (H_2_BDC), 2-amino terephthalic acid (ATA), and sodium hydroxide pellets (NaOH) were purchased from Sigma-Aldrich and used as received. Polyester was obtained from Elastic Fibers of America, and the 1500-thread-count (TC) cotton was purchased from Royal Egyptian Bedding. A Northwood Calliger clothes wringer was purchased from Amazon and used as the roller.

### Synthesis of Node Solution for UiO-66

The MOF precursor solutions (node and linker) were synthesized based on the work of Huelsenbeck et al.^29^ For the node solution of UiO-66, 400 mL deionized (DI) water, 100 mL acetic acid, and 43.92 g of ZrOCl_2_·8H_2_O were added to a 1 L glass Pyrex bottle sealed with hand-tightened screw-top caps and sonicated for 10 min. The solution was then heated in a convection oven at 70 °C for 2 h. The solution was cooled to room temperature and used as the node solution for UiO-66.

### Synthesis of Linker Solution for UiO-66

18.52 g of NaOH and 12.3 g of H_2_BDC were added to 500 mL of DI water. The solution was then stirred at room temperature until it was completely dissolved, and the resulting solution was used as the linker solution for UiO-66.

### Synthesis of Node Solution for UiO-66-NH_2_

In a 1 L Pyrex glass bottle, 161 g of ZrOCl_2_·8H_2_O was added to a mixture of 300 mL DI water and 125 mL acetic acid. The solution was then sonicated for 10 min and heated in the oven at 70 °C for 2 h. The solution was then cooled to room temperature and used as the node solution for UiO-66-NH_2_.

### Synthesis of Linker Solution for UiO-66-NH_2_

To 400 mL of DI water, 14.53 g of NaOH and 10.71 g of ATA were added and mixed via stirring at room temperature. After complete dissolution of the linker, the solution was used as the linker solution for UiO-66-NH_2_.

### Fabrication of UiO-66/UiO-66-NH_2_ Coated Fabrics

To fabricate large-area fabrics with either UiO-66 or UiO-66-NH_2_, the node and linker solutions of the respective MOF were first poured into separate containers, as shown in Figure 1b. Then, the fabric (9 in. × 6 in.) was dipped in the node solution until the entire surface of the fabric was soaked with the node solution. Then, the node-soaked fabric was passed through the roller, where excess liquid was squeezed out. The fabric was then dipped in the linker solution, where the MOF was synthesized on its surface. For the UiO-66-polyester fabrics, the node-soaked fabrics were dipped until soaked (10–15 s), consistent with our previously reported protocol.^29^ For the UiO-66-NH_2_ on polyester and cotton fabrics, a longer soak time of ∼5 min in the linker solution per SQD cycle was used to ensure complete linker coordination. The fabric was again passed through the roller to squeeze out excess solution, completing one SQD cycle. Throughout the SQD process, the node and the linker solution, as well as the roller, were maintained at room temperature. A total of 8 SQD cycles were performed. The MOF-fabric was then dried in the oven at 120 °C for 2 h. To remove any loosely bound MOF particles, the dried MOF-fabric was sonicated in water and then dried again at 120 °C before further characterization.

### Characterizations

X-ray diffraction (XRD) patterns were obtained using a Malvern-Panalytical Empyrean diffractometer with Cu Kα radiation at 45 kV and 40 mA. Scanning electron microscopy (SEM) and energy dispersive X-ray spectroscopy (EDS) images were obtained using a FEI Quanta 650 scanning electron microscope.

### Filtration Measurement

The filtration efficiency and breathability of the UiO-66-polyester MOF-fabrics were evaluated at Nelson Laboratories as specified in ASTM F3502–24. The mask samples were sealed onto a metal plate and mounted in a chamber within a TSI CERTITEST Model 8130 automated filter tester capable of measuring efficiencies up to 99.999%. A neutralized, polydispersed aerosol of sodium chloride (NaCl) with a count median diameter of 0.075 ± 0.020 μm (mass median diameter of 0.26 μm) was passed through the entire test article at a flow rate of 85 ± 4 L/min. The performance of the masks was determined by comparing the concentration of NaCl particles penetrating the masks with the concentration entering the masks. The following formula was used to calculate the filtration efficiency of the masks:

$$\mathrm{Filtration}\;\mathrm{Efficiency}\;\left(\%\right)=\left(\frac{C_{u}-C_{d}}{C_{u}}\right)\times 100\%$$

where *C*_u_ = Particle concentration upstream (before the mask); *C*_d_ = Particle concentration downstream (after the mask).

### Cytotoxicity Measurement

The cytotoxicity of the UiO-66-polyester was determined using the Minimum Essential Media (MEM) elution test. MOF-fabric samples, along with positive (natural rubber latex) and negative (polypropylene pellets) controls, were extracted in 1X MEM with 5% bovine serum for 24 h at 37 ± 1 °C with agitation. The test extracts were held at room temperature for less than 4 h before testing. L-929 fibroblast cells were seeded in multiple-well cell culture plates and incubated until 80% confluent. The test extracts were then added to the cell monolayers in triplicate and were incubated at 37 ± 1 °C for 48 ± 3 h. Following incubation, cell monolayers were inspected under a microscope for morphological changes and graded on a scale of 0–4, where 4 indicates severe cytotoxicity with nearly complete cell lysis, and 0 indicates no cytotoxic response. The final cytotoxicity score was determined by averaging the triplicate wells. Control responses verified the assay’s validity: the positive control had a reactivity grade of 4, while the negative and medium controls showed no reactivity (grade 0).

### CWA Degradation

Composites were evaluated for CWA removal using a dose-extraction method. Approximately 30 mg of composites were placed in 2 mL autosampler vials, dried overnight at ∼65 °C, and then humidified at 50% relative humidity (RH) at 25 °C. Chemical agents GD, HD, and VX were dosed at a ratio of 1 μL agent to 10 mg composite. After 24 h, the autosampler vials were immersed in a container of crushed dry ice to quench the reaction and extracted with 1.5 mL of appropriate solvents (acetonitrile for GD, chloroform for HD, and isopropyl alcohol for VX). The mixture was vortexed for 30 s and then filtered with a 0.45 μm nylon syringe filter. The resulting filtrate was analyzed using an Agilent 5973 gas chromatograph equipped with a mass selective detector. The amount of chemical reacted was determined based on the difference between the 3 μL standards and the amount detected in the filtrate.

### Chlorine Microbreakthrough

Microbreakthrough testing was conducted to determine the capacity of composites for chlorine gas. Composites were packed in 4 mm diameter fritted glass tubes. A ballast containing pressurized chlorine was delivered via a mass flow controller and mixed with diluent air to achieve a concentration of 2,000 mg/m^3^ and then fed through the composite bed. The effluent was continuously monitored until full saturation, and the loading was calculated by subtracting the integral of the effluent from the integral of the feed breakthrough curves.

## Results and Discussion

Large-area MOF-fabrics (9″ × 6″) were fabricated using the SQD process, as shown in Figure 1b. Because our previous study demonstrated rapid (subminute) synthesis time scales for MOFs (UiO-66 and UiO-66-NH_2_), we expected MOF formation to proceed at a similar rate on the fabric surface. A roller was used to remove excess reactants from the fabric, limiting MOF formation to the fabric surface. It is worth noting that the roller pressure is critical in the SQD process. Insufficient pressure promotes MOF growth in the interstitial space between fibers, yielding loosely bound particles, whereas excessive pressure reduces solution uptake and, consequently, MOF loading per cycle. Confining MOF growth to the fabric surface enhances interaction between the fabric and the MOF while preventing the formation of loosely bound MOF particles that could pose an inhalation risk when used as a facial covering. In addition, the roller mimics the roll-to-roll coating process used in industry, demonstrating the potential scalability of our coating process. Eight SQD cycles were chosen because the PM filtration efficiency was expected to plateau, as reported in our previous study. The MOF-fabrics were then washed and sonicated to remove any loosely bound particles, dried, and characterized.

SEM was first used to examine the surface morphology of the fabric after SQD. Unlike the pristine polyester fibers in Figure 2a, numerous small particles are present on the polyester fiber after SQD, as shown in Figure 2b and Figure S1. This surface growth resulted in a MOF loading of 4.1 ± 0.5% on the polyester after the SQD process (Table S2). To determine the elemental composition of these particles, EDS was performed on the UiO-66-polyester sample. Figure 2c shows the elemental mapping of Zr atoms on the surface of UiO-66-polyester. The uniform distribution of Zr confirms that these small particles correspond to UiO-66 crystals formed on the fabric surface. XRD was then used to confirm the formation of crystalline UiO-66 on the polyester fabric surface. As shown in Figure 2d, the XRD pattern of UiO-66-polyester matches those of the UiO-66 and polyester controls, confirming the synthesis of UiO-66 on the fabric surface. The diffraction peaks at reciprocal space (q) values of 0.52 and 0.60 Å^–1^ correspond to the diffraction from the (111) and (200) crystal planes of UiO-66.^36^

**Figure 2:**
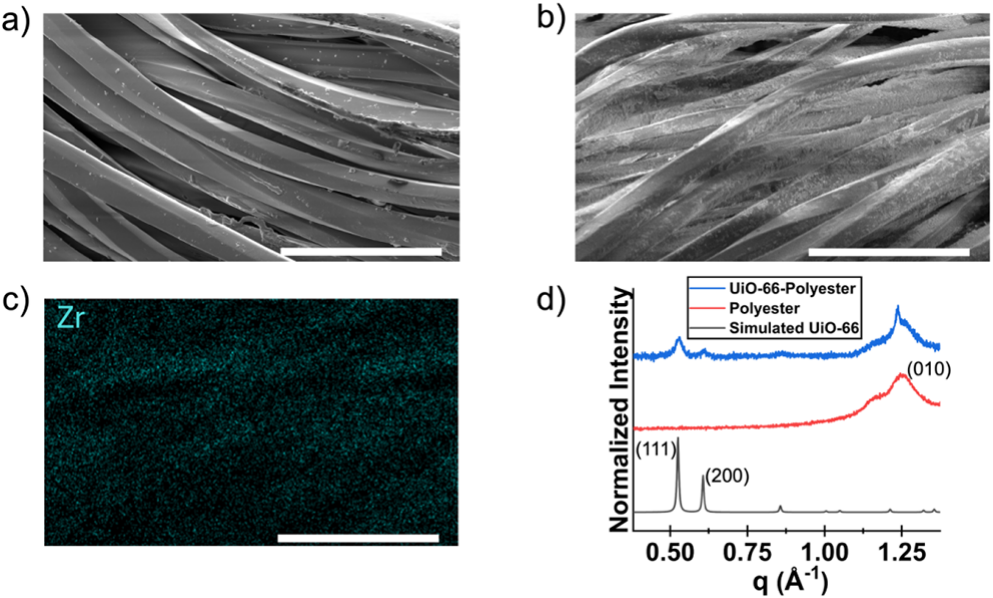
a) SEM image of polyester, b) SEM image of UiO-66-polyester, c) EDS elemental mapping of zirconium (Zr) corresponding to the SEM image in b) [Scale = 50 μm]. d) Diffraction pattern of UiO-66-polyester along with the patterns of polyester control and simulated UiO-66. The UiO-66 diffraction peaks at *q* values of 0.52 and 0.60 Å^–1^ correspond to the diffraction from the (111) and (200) crystal planes, respectively. The polyester diffraction peak at 1.26 Å^–1^ represents diffraction from the (010) plane.^35,36^

To further verify the presence of UiO-66 on the polyester fibers, N_2_ adsorption isotherms at 77K were collected for the UiO-66-polyester composite and the polyester control (Figure S2). The polyester control showed negligible N_2_ uptake with a BET surface area of 0.3 m^2^/g, whereas the UiO-66-polyester composite exhibited a BET surface area of 3.4 m^2^/g, corresponding to a normalized MOF surface area of 83 m^2^/g. This value represents approximately ∼25% retention of the intrinsic surface area of UiO-66 powder synthesized using the same rapid aqueous method by Huelsenbeck et al. (339 ± 35 m^2^/g).^29^ The lower normalized surface area may reflect uncertainty in the mass loading analysis and shielding of MOF crystal surfaces by the polyester fibers, a common issue for MOFs grown in the presence of polymer or fibrous substrates.^37–39^ Additionally, because the fibers are soaked in both precursor solutions, some MOF may have formed within the fibers rather than exclusively on their surfaces, thereby blocking MOF pores. Furthermore, the MOF loading estimated from the BET surface area has been shown to be lower than that determined gravimetrically for MOF-fabric composites.^38^ Nevertheless, the measured N_2_ uptake is greater than that of the fabric control, confirming the presence of accessible MOF porosity in the composite and providing independent verification of successful UiO-66 synthesis on the polyester fiber surface beyond the XRD and SEM results.

After confirming the synthesis of UiO-66 on the polyester surface, we sewed the MOF-fabric into a bilayer facial covering similar in size to standard masks available on the market (Figure S3). The masks were then tested externally at Nelson Laboratories for filtration efficiency and breathability in accordance with the ASTM F3502 specification. The ASTM F3502 outlines two performance levels (Level 1 and Level 2), with Level 2 indicating higher filtration efficiency and lower airflow resistance (Table S1).^22^ Within this standard, filtration efficiency is measured by passing ∼0.3 μm polydispersed NaCl aerosol through the masks at 85 L/min and comparing the NaCl concentration downstream of the mask to that of upstream. These parameters represent the most challenging test scenario: 0.3 μm corresponds to the MPPS for fibrous filter media, where diffusion- and interception-dominated capture mechanisms are least effective, and 85 L/min represents a worst-case breathing scenario for moderate-to-heavy physical activity, providing a high margin of safety in real-world use.^40^ Under these conditions, our MOF-fabric masks (*n* = 7) successfully met the Level 1 requirements for both filtration efficiency (≥20% for ∼0.3 μm particles) and breathability (≤15 mm H_2_O) (Figure 3a and Table S3). Furthermore, the MOF-fabrics showed an enhancement in filtration efficiency compared to the polyester masks (∼19.8% for ∼0.3 μm particles). The average filtration efficiency across the MOF-fabric masks was 32.5 ± 4.5% for 0.3 μm particles, with a maximum of 39.7%, confirming that the MOF-fabric masks achieve performance consistent with recognized industrial benchmarks.

**Figure 3:**
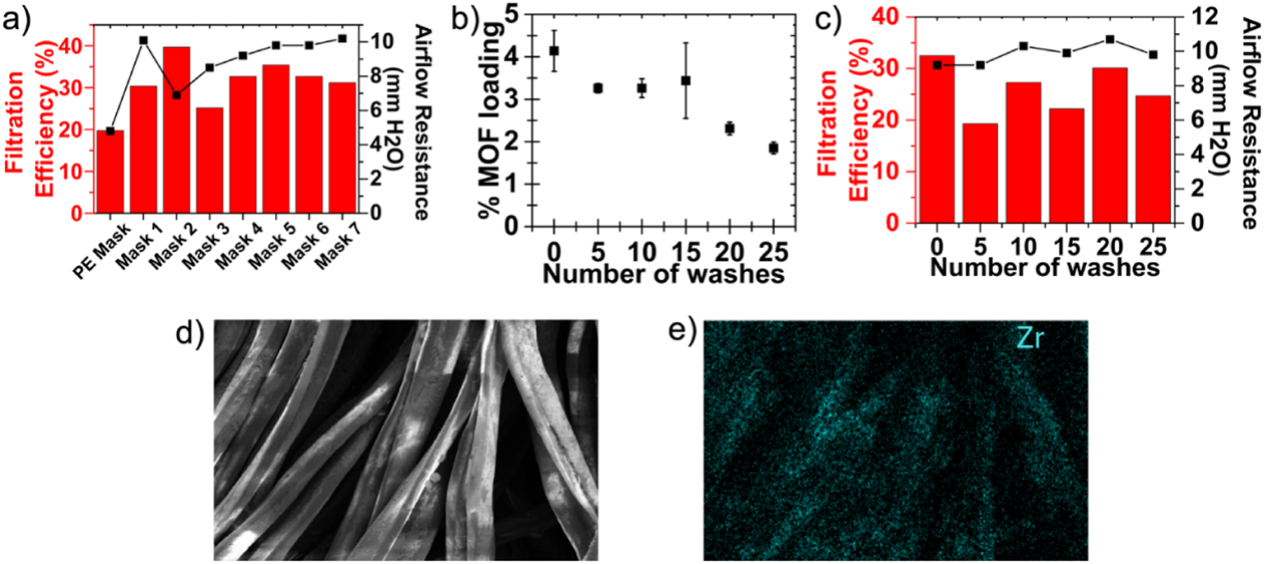
a) Filtration efficiency and breathability of bilayer UiO-66-polyester masks, b) Changes in MOF loading with the number of washing cycles, c) Filtration efficiency and airflow resistance as a function of washing cycles, d) SEM image of UiO-66-polyester after 25 washing and drying cycles, and e) EDS elemental map of Zr corresponding to the SEM image on d).

Assessing the cytotoxicity of our MOF-fabrics is crucial for their use as facial coverings, as they are intended for direct skin contact and prolonged wear. Cytotoxicity testing was also conducted at Nelson Laboratories in compliance with U.S. Food and Drug Administration (FDA) regulations (21 CFR Parts 58, 210, 211, and 820). The Minimum Essential Media (MEM) elution test, in which L-929 mouse fibroblast cells were exposed to UiO-66-polyester MOF-fabric extracts, was used to determine the cytotoxicity of the UiO-66-polyester MOF-fabric.^41^ The neat extract produced a reactivity grade of 2 (mild reactivity), while diluted extracts (1:2 to 1:16) yielded grades of ≤1, indicating no cytotoxic response (Table S4). Overall, the MOF-fabric displayed only mild cytotoxicity in its undiluted extract and no detectable cytotoxicity upon dilution. Since the United States Pharmacopeia & National Formulary (USP < 87>) and the International Organization for Standardization (ISO) 10993-5 standard define grade ≤2 as a passing score, these findings suggest that the MOF-fabrics are unlikely to cause adverse effects under realistic exposure conditions. Combined with the filtration and breathability results, the cytocompatibility profile supports the safe use of UiO-66-polyester MOF-fabrics in barrier face coverings.

To evaluate the reusability of the MOF-fabric masks, a systematic washing study was conducted. Each UiO-66-polyester layer was washed and dried up to 25 times in a household washing machine and dryer. The “0 wash” sample refers to the MOF-fabric after the initial laboratory sonication wash. A total of 15 MOF-fabric samples were tested, with three replicates for each of the 5, 10, 15, 20, and 25 washing cycles. As shown in Figure 3b, MOF loading (%) decreased progressively with increasing washing cycles. Notably, ∼2 wt % of UiO-66 remained on the fabric even after 25 cycles, indicating strong adherence of a fraction of the total MOF to the polyester fibers. From each washing stage, a bilayer mask was sewn and sent to Nelson Laboratories for filtration efficiency and breathability testing. Even after 25 washes, the UiO-66-polyester masks met ASTM F3502 Level 1 requirements, maintaining a filtration efficiency of ∼25% and an airflow resistance of ∼10 mm H_2_O (Figure 3c and Table S5). The SEM images (Figure 3d) reveal particles on the fabric surface, and EDS elemental mapping confirms the presence of Zr, verifying that these particles are UiO-66 (Figure 3e). Collectively, these results demonstrate the functional performance of the MOF-fabric masks over multiple washing cycles, highlighting their suitability for practical use as reusable barrier face coverings.

In addition to enhancing PM filtration, MOF-coated fabrics have been investigated for protective clothing applications aimed at degrading CWAs such as GD, HD, and VX. For CWA degradation, Zr-based MOFs such as UiO-66 and UiO-66-NH_2_ are particularly effective because the Zr-oxo node provides catalytic sites for hydrolysis, while the amino (−NH_2_) groups in UiO-66-NH_2_ further accelerate the reaction by facilitating proton transfer.^42–44^ Motivated by these findings, we extended our synthesis technique to fabricate UiO-66-NH_2_ on fabrics for CWA degradation. Using the SQD method, combined with a roller to mimic industrial roll-to-roll manufacturing, UiO-66-NH_2_ was successfully deposited onto polyester and cotton fabrics. To increase MOF loading, which is directly correlated with CWA degradation performance, we increased the Zr concentration in the node solution and extended the dipping time in the linker solution to approximately 5 min per SQD cycle.^45,46^ The rationale for increasing the Zr concentration is based on the fabric’s fixed liquid uptake capacity. When dipped in the node solution, the fabric takes up a limited volume of solution determined by its chemistry, porosity, and fiber surface area. By increasing the Zr concentration, a larger amount of Zr is integrated into the fiber surface within the fixed liquid volume. Upon subsequent immersion in the linker solution, the larger amount of available Zr-oxo clusters yields a proportionally larger amount of MOF per cycle, resulting in higher overall MOF loading after 8 SQD cycles. Furthermore, the extended linker soak time of ∼5 min provides sufficient time for the organic linker to coordinate to the increased number of available Zr centers on the fiber surface, maximizing MOF formation per cycle. This strategy resulted in UiO-66-NH_2_ loadings of 15 wt % on polyester and 31 wt % on cotton fabrics. The characteristic diffraction peaks at q values of 0.52 Å^–1^ and 0.60 Å^–1^ confirmed the formation of UiO-66-NH_2_ on both polyester and cotton (Figure 4b). Furthermore, SEM imaging revealed the presence of particles on the fibers, while elemental mapping of Zr confirmed their uniform distribution across the fabric surface (Figure 4c and d).

**Figure 4:**
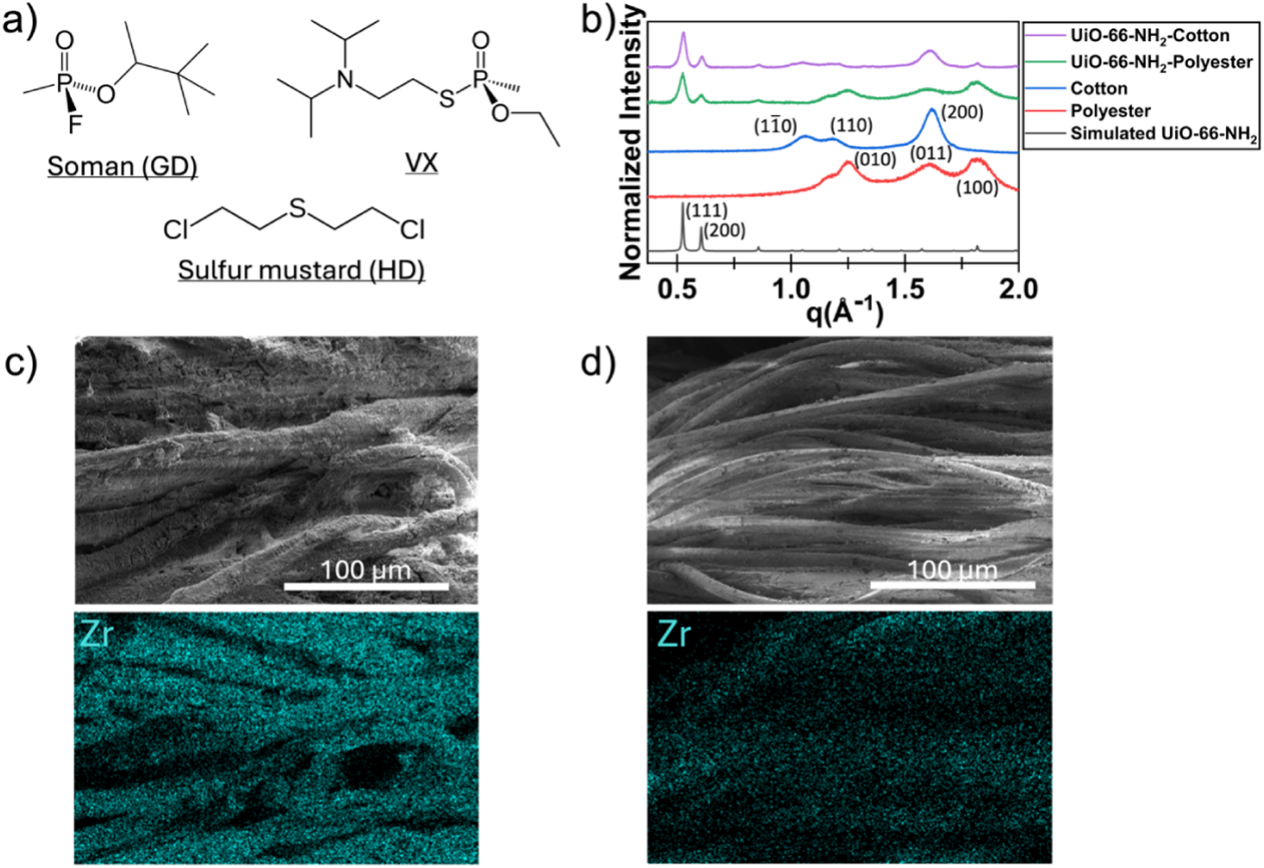
a) Molecular structure of different CWAs, b) XRD pattern of fabrics coated with UiO-66-NH_2_ along with the control fabrics, c,d) SEM images and EDS elemental mapping of zirconium (Zr) of c) UiO-66-NH_2_-cotton and d) UiO-66-NH_2_-polyester.

To assess the porosity of UiO-66-NH_2_ grown on polyester and cotton, N_2_ adsorption isotherms were collected for the polyester and cotton controls, as well as the UiO-66-NH_2_-polyester and UiO-66-NH_2_-cotton composites (Figure S4). Polyester and cotton controls showed negligible N_2_ uptake compared to MOF-fabrics. Polyester had a BET surface area of 0.3 m^2^/g while cotton had a surface area of 0.4 m^2^/g. UiO-66-NH_2_-polyester and UiO-66-NH_2_-cotton had BET surface areas of 58 m^2^/g and 104 m^2^/g, respectively. UiO-66-NH_2_-cotton had a higher surface area due to higher MOF loading. When normalized based on the MOF loading, the accessible MOF surface area was approximately 388 m^2^/g for UiO-66-NH_2_-polyester and 337 m^2^/g for UiO-66-NH_2_-cotton, representing 47–54% retention of the intrinsic surface area of aqueously synthesized UiO-66-NH_2_ powder (717 ± 128 m^2^/g), showing reduction of the surface area relative to the free powder, similar to the case of the UiO-66 samples.^37^

The UiO-66-NH_2_-fabrics were then tested for the removal of CWAs (GD, HD, and VX) using the dose-extraction method as discussed in the Methods section. The amount removed for these chemicals by the UiO-66-NH_2_-polyester and UiO-66-NH_2_-cotton composites is summarized in Table 1. Both composites removed ∼80% of GD after 24 h, while the cotton composite showed significantly better HD and VX removal than the polyester composite. These trends align with the greater amount of MOF in the cotton composites. Overall, the materials demonstrated excellent CWA removal for fiber-based systems. Chlorine gas was also dosed onto materials to assess the potential of filters to remove low levels of toxic chemicals. As with the CWAs, the cotton composite showed better overall chlorine removal than the polyester composite (Table 2 and Figure S5). We calculated a hypothetical loading that the composites should achieve based on the amount of MOF in the composite and the chlorine uptake of pure UiO-66-NH_2_ (∼5 mol/kg). Both composites showed slightly better than the hypothetical loading, indicating that the MOFs are active and easily accessible on the fibers.

**Table 1:** CWA Reactivity of Composites

	% Reacted in 24 h		
Material	GD	HD	VX
UiO-66-NH_2_ on Polyester	80	34	52
UiO-66-NH_2_ on Cotton	82	58	89

**Table 2:** Cl_2_ Uptake of Composites

Material	Cl_2_ loading (mol/kg)	Hypothetical loading (mol/kg)
UiO-66-NH_2_ on Polyester	0.84	0.77
UiO-66-NH_2_ on Cotton	1.88	1.58

## Conclusions

In summary, this work demonstrates a scalable strategy for fabricating MOF-fabrics relevant to both PM filtration and CWA degradation. By combining roll-to-roll coating with the SQD process, we successfully synthesized Zr-based MOFs, including UiO-66 and UiO-66-NH_2_, on polyester and cotton surfaces. Large-scale (9″ × 6″) UiO-66-polyester was synthesized and evaluated according to the ASTM F3502 standards, exhibiting an average PM filtration efficiency of 32.5 ± 4.5% with a breathability of ∼9.2 ± 1.2 mmH_2_O, satisfying the level 1 performance criteria. Furthermore, the UiO-66-polyester masks retained their filtration performance even after 25 washing and drying cycles, highlighting their reusability. The MEM elution test confirmed the noncytotoxicity of the UiO-66-polyester, making it safe for use as a barrier face covering. Beyond PM filtration, enhanced degradation of CWAs, such as GD, HD, and VX, was also achieved using UiO-66-NH_2_-polyester and UiO-66-NH_2_-cotton fabrics. UiO-66-NH_2_-cotton achieved 89% and 82% conversion of VX and GD, respectively, after 24 h. Overall, this study establishes a simple and scalable route for MOF-fabric production and highlights their potential as next-generation smart textiles and multifunctional protective materials.

## Supplementary Material



## Notes

Table showing the performance criteria for different levels of ASTM F3502 certification, a table with the change in MOF loading with the number of washing cycles, SEM images of the MOF-fabric, a picture of the bilayer masks as tested, N_2_ adsorption isotherms, tables showing the cytotoxicity results, filtration efficiencies, and breathability of UiO-66-polyester fabrics, including the change in performance as a function of washing cycles, Cl_2_ breakthrough curves (DOCX)
