# Supplementary material for: Roll-To-Roll Coated Metal–Organic Framework (MOF)-Fabric-Based Filters for Particulate Matter Filtration and Chemical Warfare Agent Degradation
Source: ACS Appl Mater Interfaces. 2026 May 27;18(22):31234–41. doi: 10.1021/acsami.6c03444 (PMC13266710; doi:10.1021/acsami.6c03444)
Supplement: Supplementary file 1 [file am6c03444_si_001.docx]

Supplemental Information (SI)

Roll-To-Roll Coated Metal-Organic Framework (MOF)-Fabric-Based Filters for Particulate Matter Filtration and Chemical Warfare Agent Degradation

Ankit Dhakal^1^, Lauren Hernon^1^, Sangeun Jung^1^, Emily Beyer^1^, Sean Robinson^1^, Luke Huelsenbeck^1^, Prince Verma^1^, Bala Mulloth^2^, Gregory W. Peterson^3^ and Gaurav Giri^1^*

^1^ Department of Chemical Engineering, University of Virginia, Charlottesville, VA, 22904, USA

^2^ Frank Batten School of Leadership and Public Policy, University of Virginia, Charlottesville, VA, 22904 USA

^3^ U. S. Army Combat Capabilities Development Command Chemical Biological Center, Aberdeen Proving Ground, MD, 21010, USA

KEYWORDS: metal-organic framework, UiO-66, filtration, chemical warfare agent degradation

* Corresponding Author: [gg3qd@virginia.edu](mailto:gg3qd@virginia.edu)

**Table S1:** ASTM F3502 Standard for barrier face coverings^1^

| Testing Criteria | Level 1 | Level 2 |
| --- | --- | --- |
| Fitration (NaCl Aerosol ~0.3 $\mu m$) | $\geq$20 % | $\geq$ 50% |
| Breathability (Airflow resistance) | $\leq$15 mmH_2_O | $\leq$6 mmH_2_O |


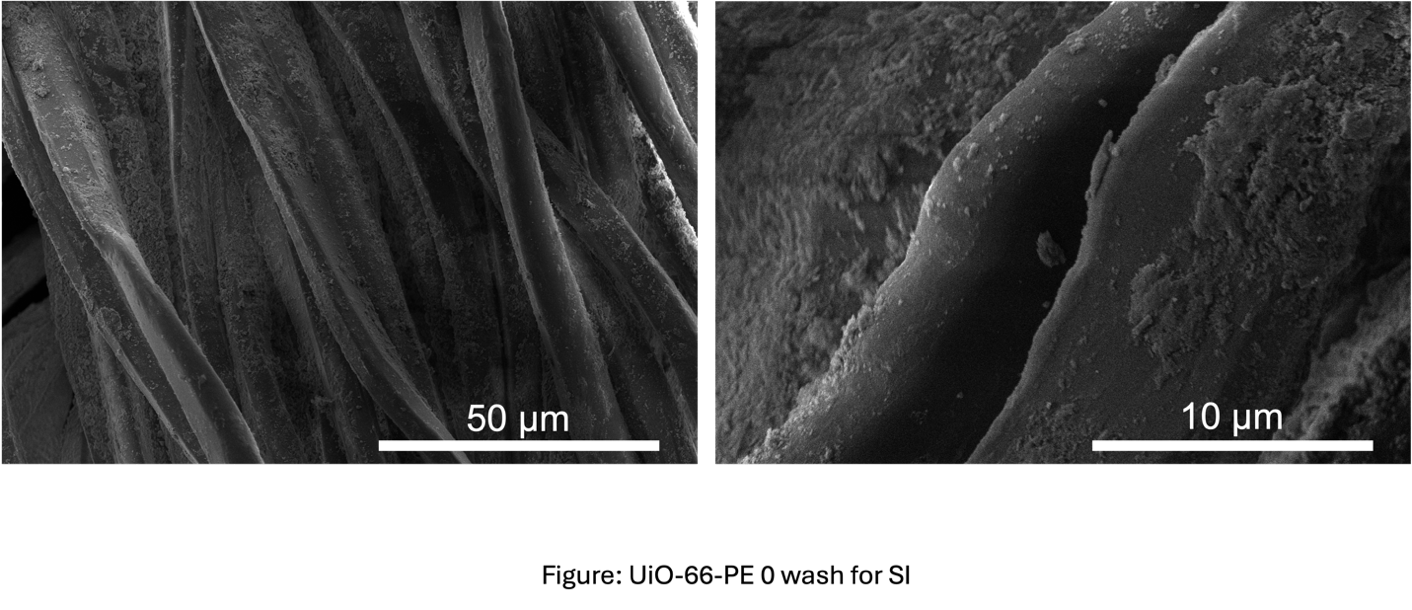


**Figure S1**: SEM images of UiO-66-Polyester.

**Table S2**: UiO-66 loading on Polyester fabric after 8 SQD cycles and washing up to 25x.

| Number of washes | MOF loading (%) |
| --- | --- |
| 0 | 4.1$\pm$0.5 |
| 5 | 3.3$\pm$0.1 |
| 10 | 3.3$\pm$0.2 |
| 15 | 3.4$\pm$0.9 |
| 20 | 2.3$\pm$0.2 |
| 25 | 1.9$\pm$0.1 |

The % MOF loading was calculated using the following formula:

$$\frac{(Mass of MOF-fabric after initial washing via sonication-Initial mass of fabric control)}{(Initial mass of fabric control)} x 100 \%$$

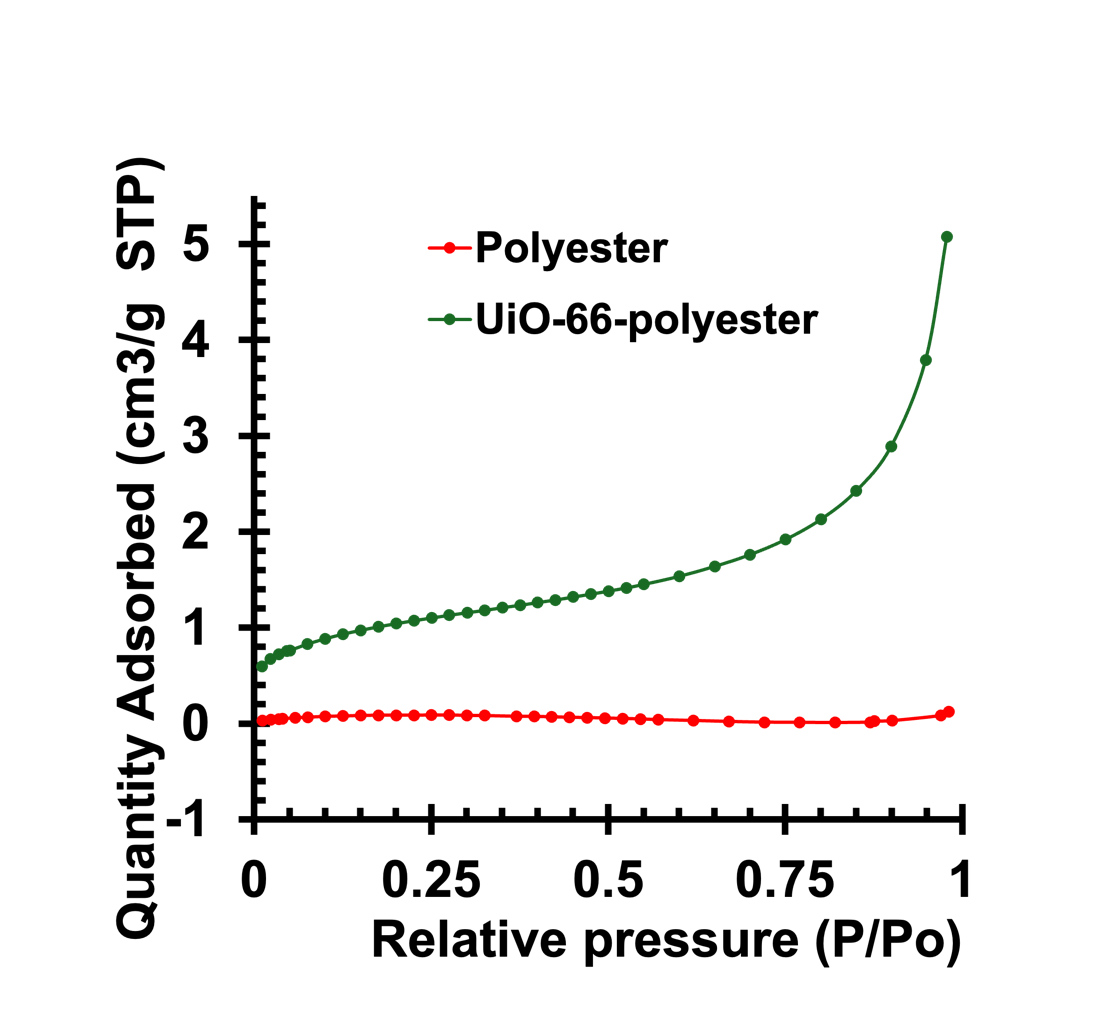


**Figure S2:** N_2_ adsorption isotherm of UiO-66-polyester fabrics.


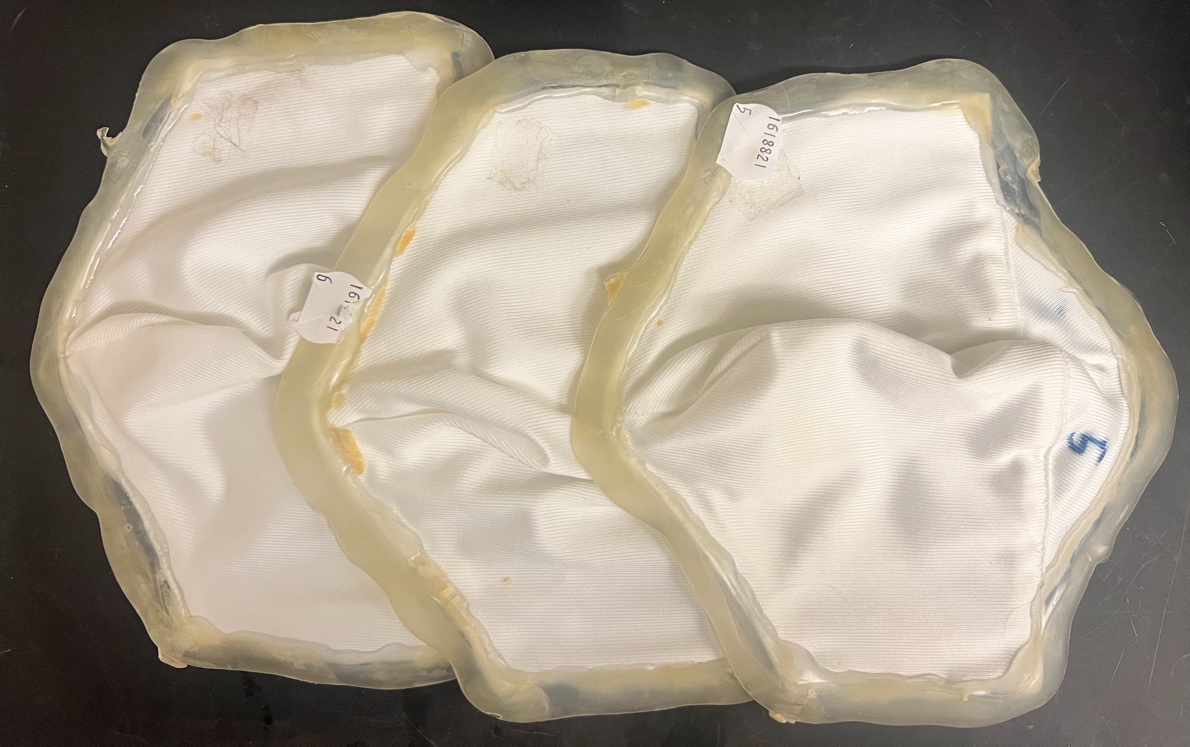


**Figure S3:** Bilayer UiO-66-Polyester Masks

**Table S3:** Filtration efficiency and breathability of the bilayer masks.

| Sample | Filtration Efficiency (%) | Airflow Resistance (mm H_2_O) |
| --- | --- | --- |
| Polyester control mask | 19.8 | 4.8 |
| Mask 1 | 30.4 | 10.1 |
| Mask 2 | 39.7 | 6.9 |
| Mask 3 | 25.2 | 8.5 |
| Mask 4 | 32.7 | 9.2 |
| Mask 5 | 35.4 | 9.8 |
| Mask 6 | 32.7 | 9.8 |
| Mask 7 | 31.2 | 10.2 |
| Average across UiO-66-PE masks | 32.5 $\pm$ 4.5 | 9.2 $\pm$ 1.2 |

**Table S4**: Cytotoxicity results of mask extracts by MEM Elution test (reactivity grades ≤ 2 = Pass)

| Dilution | Average Score | Results Pass/Fail |
| --- | --- | --- |
| Neat | 2 | Pass |
| 1:2 | 1 | Pass |
| 1:4 | 0 | Pass |
| 1:8 | 0 | Pass |
| 1:16 | 0 | Pass |

0 = No cell lysis, no reactivity; 1 = Less than or equal to 20 % rounding, occasional lysed cells, slight reactivity; 2 = 20-50 % rounding, no extensive cell lysis, mild reactivity; 3 = 50-70 % rounding and lysed cells, moderate reactivity; 4 = Nearly complete destruction of the cell layers, Severe reactivity.

**Table S5:** Change in filtration efficiency and breathability with the number of washing cycles.

| Number of washing cycles | Filtration Efficiency (%) | Airflow Resistance (mm H_2_O) |
| --- | --- | --- |
| 0 | 32.5 | 9.2 |
| 5 | 19.3 | 9.2 |
| 10 | 27.3 | 10.3 |
| 15 | 22.2 | 9.9 |
| 20 | 30.1 | 10.7 |
| 25 | 24.7 | 9.8 |


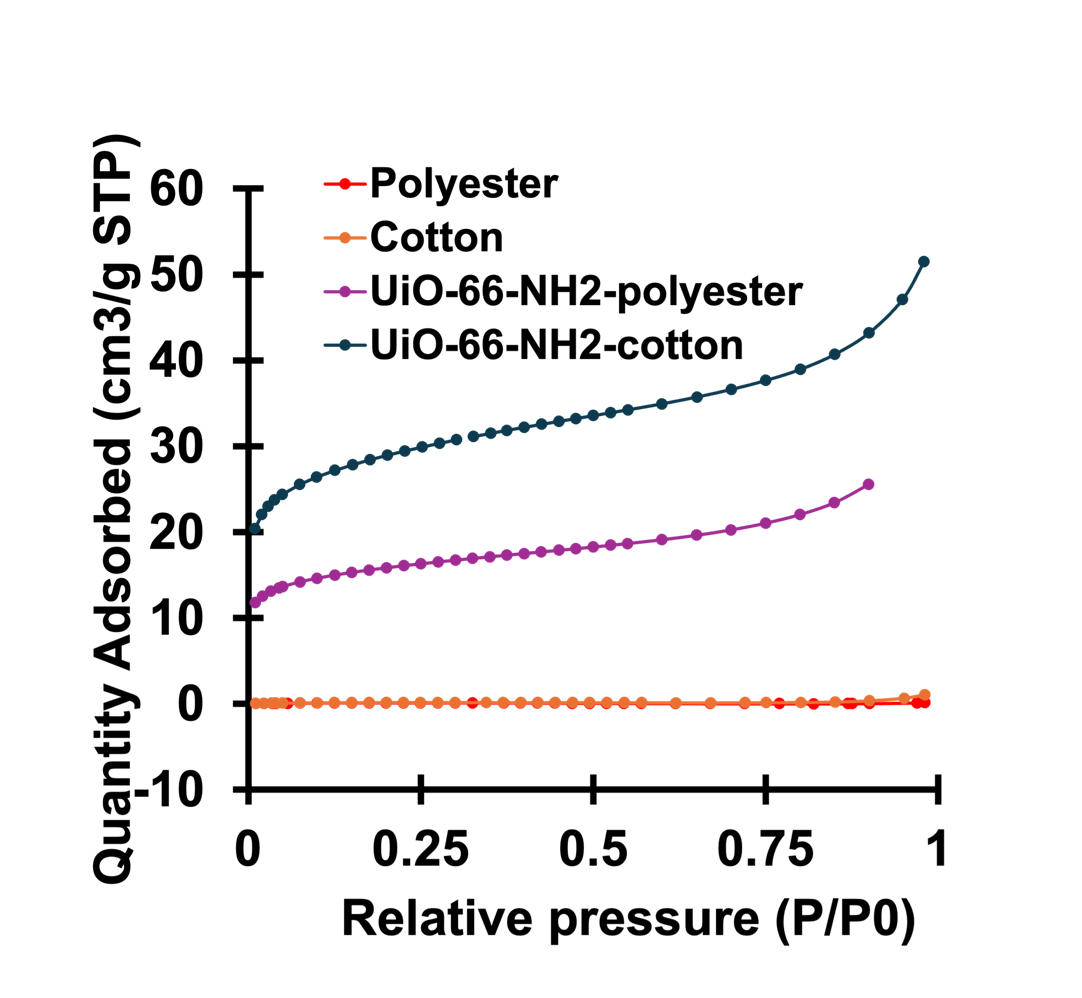


**Figure S4:** N_2_ adsorption isotherm of UiO-66-NH_2_-fabrics.

**Figure S5:** Chlorine breakthrough curves of MOF-fabric


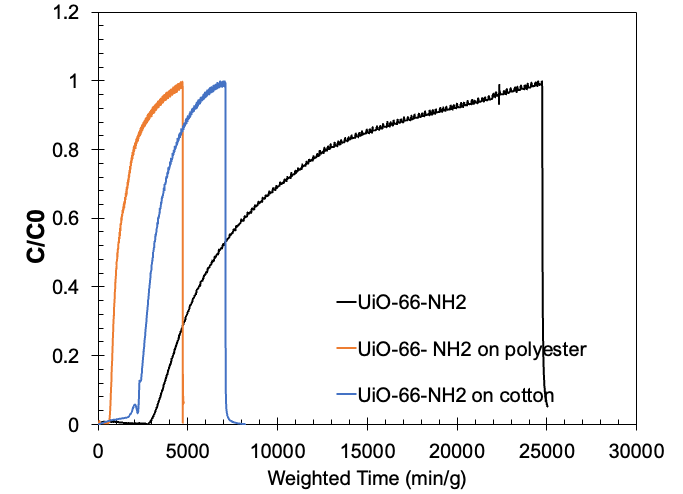


**References:**

(1) F3502-21 Standard Specification for Barrier Face Coverings, 2021.
